# The protective effect of activating Nrf2 / HO-1 signaling pathway on cardiomyocyte apoptosis after coronary microembolization in rats

**DOI:** 10.1186/s12872-017-0704-1

**Published:** 2017-10-24

**Authors:** Jiabao Liang, Lang Li, Yuhan Sun, Wenkai He, Xiantao Wang, Qiang Su

**Affiliations:** Department of Cardiology, The First Affiliated Hospital of Guangxi Medical University, Nanning, Guangxi Zhuang Autonomous Region 530021 China

**Keywords:** Nuclear factor erythroid 2-like, Heme oxygenase-1, Coronary microembolization, Apoptosis

## Abstract

**Background:**

Myocardial apoptosis is closely related to myocardial injury caused by coronary microembolization (CME).Nuclear factor erythroid 2-like (Nrf2) has been taken into account as an inhibitor of apoptosis in various tissues. Thus, this research aims to investigate which part Nrf2/HO-1 signaling pathway plays in myocardial apoptosis process following the effect of CME on rats.

**Methods:**

Separate 40 rats then form them into a group of shame, a group of CME, a group of CME plus AAV-Nrf2(AAV-Nrf2 (CME) group) and a group of CME plus AAV-control (AAV-control (CME) group) stochastically and averagely. Rat CME was established by injecting into the left ventricular chamber, with or without pretreatment of adeno-associated virus Nrf2 (AAV-Nrf2). Echocardiological measurements, using Terminal-deoxynucleoitidyl Transferase Mediated Nick End Labeling (TUNEL) to stain, conducting Quantitative PCR in real time (RT-PCR) as well as Western blotting to evaluate the impacts of them functionally, morphologically and molecularly in CME.

**Results:**

Nrf2 decreased in cardiomyocytes after CME. Upregulation of Nrf2 inside an organism through AAV connect to improving the function of heart as well as attenuating myocardial apoptosis, following the restrain of proapoptotic mRNAs and proteins like caspase-3, caspase-9 and bax expressing as well as the increase of antiapoptotic mRNA and proteins like HO-1 and bcl-2 expressing.

**Conclusion:**

Activation of Nrf2/HO-1 pathway can improve CME-induced cardiac dysfunction effectively and also reduce the myocardial apoptosis.

## Background

Coronary microembolization (CME) is among those vital causes of dysfunctional heart without atherosclerotic obstacle inside the epicardial coronary artery. Tens of years ago, CME was discovered at postmortem for the first time in patients who died as a result of a unexpected cardiac events [1]. Nevertheless, CME, following thrombolytic treatment and coronary disturbances for patients suffering a coronary thrombus, is becoming more regularly [2], or a remote arteriole thrombus in a spontaneous lysis. CME may cause myocardial perfusion-contraction mismatch, arrhythmias, inflammation and micro-infarction and so on, which can possibly result in harmful or fatal consequences [3–8]. CME is a fundamental reason of non-blocked coronary artery illness. Numerous investigations have evidenced the underlying mechanisms of CME-induced myocardial damage as well as relieve the deleterious effects of CME. A considerable amount of studies demonstrated that cardiomyocyte apoptosis was a vital part of the mechanism of CME-induced myocardial damage [9–11], and extra modulatory mechanisms still need to be explained because of the overall complexity of myocardial injury.

Heme oxygenase-1(HO-1) is a phaseIIdefense enzyme which has potent antiapoptotic as well as antioxidative stress effects. In those HT22 cells, HO-1 is over expressed which confer antiapoptotic protective effects against H/R [12]. Nuclear factor erythroid 2-like (Nrf2) is a primary element of transcription. It adjusts numerous ways of apoptosis in a cell [13]. Recent studies have paid more attention to the Nrf2/HO-1 approach that is a key part in the apoptosis and that regulates and lowers the cardiomyocyte apoptosis [14].

The purpose of the current research was in order to determine the part of Nrf2/HO-1 in CME-induced myocardial apoptosis. Here, we focused on the interactions of Nrf2/HO-1 pathway with the bcl-2, bax, caspase3 and caspase9 in CME-induced myocardial apoptosis. The study suggested that Nrf2/HO-1 was a major part in CME-induced myocardial apoptosis and activation of Nrf2/HO-1 pathway could remarkably restrain apoptosis in CME-induced myocardial as well as enhance the function of heart. We supplied a possible CME-induced myocardial apoptosis mechanism.

## Methods

### Animal preparation

Sprague-Dawley rats (250-300 g) were gained from the Animal Center of the Guangxi Medical Collage which is in Nanning, China. During every phase of this experiment, the rats were raised with manipulated light status, moisture, with clean water and feed provided unlimited. All the procedures were authorized and supervised by the Animal Experiment Ethics Committees of Guangxi Medical Collage.

### Design, synthesis as well as transfection of AAV-Nrf2

The adeno-associated virus Nrf2(AAV-Nrf2) gene sequence was located in GeneBank (Gene ID: 83619) and compounded by Hanbio Biotechnology Co (Shanghai, China). Control AAV was also bought from this company. A dose of 10╳11E in all injected by tail veins was mixed with saline for each rat.

### Creation of a CME model and grouping

CME model was generated as described by Li L et al. previously [15]. Forty rats were separated then formed into 4 groups, containing a sham group, CME group, CME plus AAV-Nrf2 group (AAV-Nrf2(CME) group) and CME plus AAV-control group (AAV-control (CME) group), with 10 rats in each one of them. AAV-Nrf2(CME) group and AAV-control (CME) group both received 2-week transfection of AAV-Nrf2 or AAV-control virus. And then, the two groups received the following operation: put simply, a sternotomy in the middle the second and third intercostal was undertaken. The pericardium was unfolded and the increasing aorta was totally revealed. The plastic microspheres (3000 microspheres per rat, diameter of 42 μm, Biosphere Medical Inc., Rockland, USA) dissolved in 0.1 mL NS were injected into the ventricular (LV) chamber on the left and meanwhile clamping the ascending aorta with the use of the hemostatic forceps during the 10 s. Rats in the control group received 0.1 ml normal saline (PubChem CID: 5234) merely as the sham group did.

### Echocardiography study

The left ventricle was detected applying echocardiography which was used to examine the left ventricle of rats, which were lightly anesthetized by injecting 10% chloral hydrate (1-2 ml/kg) into an enterocoelia. In a word, an S12 transducer was used to be put on the left side and anterior position of chest wall to gain the left ventricle end-diastolic dimension (LVEDd), left ventricle ejection fraction (LVEF), cardiac output (CO) as well as fractional shortening (FS). A Philips Sonos 7500 system (Philips, Andover, USA) was used then all echocardiography was carried on by a veteran physician. Evaluating cardiac function three times, and the average values of the three figures were taken.

### Myocardial micro-infarct size measurement

HBFP stain was applied to detect myocardial ischemia in an early stage or infarct area. The dyed yellow or brown myocardial tissue was normal while the red tissue was ischemia or necrotic. A DMR-Q550 pathological picture analyzer (Lei, Germany) analyzed The HBFP-stained sections before. In short, 10 optical areas in the microscope (magnification, ×100) were casually selected from every part and Leica Qwin analysis software was used to observe. The formula- ischemic area/total area × 100% was used to calculate the relative ischemic area [16].

### Using TUNEL assay to examining myocardial apoptosis

This process was grimly conformed to the instructions of Kit specification. Apoptotic nuclei showed yellow-brown while the normal colour is light blue (TUNEL positive), and a whole amount of 10 non-overlapping areas (magnification,×400) from every slice which was observed stochastically [17]. The myocardial apoptotic ratio = apoptotic number/entire cell number × 100% was to be used.

### Quantitative Polymerase Chain Reaction in real time (RT-PCR)

We extracted the entire RNA from the cardiac tissue of rats with TRIzol® Reagent (Takara, Japan) 6 h after CME operation. Reverse transcription RNA (1 μg) was performed applying a reverse transcriptase kit (Takara, Japan) referring to the instructions provided by the manufacturer. Reactions involved 2 μL cDNA, 2 μL primers and 12.5 μL SYBR Premix Ex Taq II, and put into a ultimate volume of 20 μL of water. Real time-PCR was undertaken the next phase situations: ten-min initial denaturation at 95 C following 15-s 40 phases of amplification with 95 C as well as 1-min 60 °C. PCR in real time was applied with the following primers: 5′-GCTGCCATTAGTCAGTCGCTCTC -3′ and 5′- ACCGTGCCTTCAGTGTGCTTC-3′ for the rat nuclear relative factor 2-like (Nrf2), 5′- TTAAGCTGGTGATGGCCTCC -3′ and 5′- GTGGGGCATAGACTGGGTTC-3′ for the rat heme oxygenase1(HO-1), and 5′-ACTGCTTCCCAGACCCACA-3′ and 5′-CGAGACCTTGGAACACAGAGAA-3′ for caspase9, and 5′-GCAGCAGCCTCAAATTGTTGAC-3′ and 5′-TGCTCCGGCTCAAACCATC-3′ for caspase3, and 5′-AGACACCTGACCTTGGA-3 and 5′-TTGAAGTTGCCATCAGCAAACA-3 for bax, and 5′-AGACACCTGACCTTGGA-3 and 5′-TTGAAGTTGCCATCAGCAAACA-3 for bcl-2, and 5′-GAGATTACTGCCCTGGCTCCTA-3′ and 5′-CATCGTACTCCTGCTTGCTGAT-3′ for β-actin. The dissociation curve was analyzed to confirm specific amplification. The amplification of the overall cDNA samples were divided into 3 portions and standardized compared to a triplicate of β-actin in this dish. The expression of the data was 2–∆∆Ct.

### Western blotting

Proteins were extracted from tissue samples from 6 h after CME operation. Subsequently, the protein (50 μg) was quantified using a BCA assay (Solarbio, Beijing, China) and was loaded onto a 10% SDS-PAGE gel for electrophoresis and then transferred into a PVDF membrane (Millipore). Blocking with 5 % non-fat milk in TBS at the temperature in a room for 1 h, following the incubation of the membranes with a 1:500-10,000 dilution of primary antibody (anti-Nrf2, anti-HO-1, anti-caspase-3, anti-caspase-9, anti-bax, as well as anti-bcl-2; Abcam, UK) in buffer overnight at 4 °C, after washing 3 times in TBS, the incubation happened with a 1:10,000 dilution of fluorescent anti-rabbit secondary antibody (Abcam, UK) in buffer at the temperature in the room for 2 h. The immunoreactive bands were observed by exposing the blots in an Odyssey infrared imaging system (LI-COR).

### ELISA

To measure the expression of cTnI in serum, blood samples from each rat were collected for ELISA 6 h after CME operation based on the instructions provided by the manufacturer.

### Preparation of the tissue samples

Ten rats per group were killed under anesthesia 6 h after CME operation. Blood samples were collected and used for enzyme linked immunoassay (ELISA). Heart tissue was harvested. Four tissue samples were fixed in 4% paraformaldehyde (PubChem CID: 62081) for 24 h for preparation of paraffin sections and the myocardial infarct size was detected by HE staining and hematoxylin-red complex picric acid (HBFP) staining. The apoptosis of myocardium was detected by TUNEL staining. Three hearts were preparation for Real time-PCR. The remaining heart tissue was used for the preparation of Western blotting. All tissue samples were prepared in an identical manner.

### Statistical analysis

Data was analyzed by SPSS v18.0 (Chicago, Illinois, USA). Continuous variables are the mean ± SD. We applied one-way ANOVA (Analysis of Variance) to contrast differences among groups. Categorical variables were reported as frequencies and percentages (%), and differences between groups were examined by Fisher exact examination or chi-square examination. *P* < 0.05 was considered statistically remarkable.

## Result

### Groups of animal

No significant discrepancies statistically (*P* > 0.05) in weight of the body or rate of the heart were testified among these four groups (Table 1).

**Table 1:** The weights and heart rates of four groups before operation. There were not any statistically significant differences among four groups

Groups	Sham	CME	AAV-Nrf2	AAV-control
Weight (g)	251.2 ± 10.3	253.2 ± 12.2	249.9 ± 11.3	250.8 ± 9.3
Heart rate (bpm)	463 ± 20	460 ± 27	458 ± 32	462 ± 24

Data is shown as Mean ± SD

*Nrf2* nuclear factor erythroid 2-like, *AAV* adeno-associated virus

^a^ *P* < 0.05 contrasted with sham

^b^ *P* < 0.05 contrasted with CME or AAV control

### HBFP staining and HE staining

**Fig. 1:**
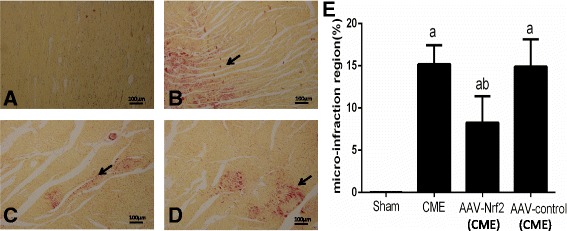
Microinfarction detected with HBFP staining (×100), bar = 100 μm. The normal myocardial staining was yellow, while the stain of ischemic myocardium was reddish. Arrows reveal microinfarction focus. **a** Sham; **b** CME; **c** AAV-Nrf2(CME); **d** AAV-control (CME). **e** The percentage of microinfarction area. AAV-Nrf2(CME): rats injected Nrf2 undergone CME operation; AAV-control (CME): rats injected AAV-control undergone CME operation; CME: coronary microembolization; Nrf 2: nuclear factor erythroid 2-like; AAV: adeno-associated virus. Data is shown as Mean ± SD. a: *P* < 0.05 contrasted with sham; b: *P* < 0.05 contrasted with CME or AAV control (CME)

**Fig. 2:**
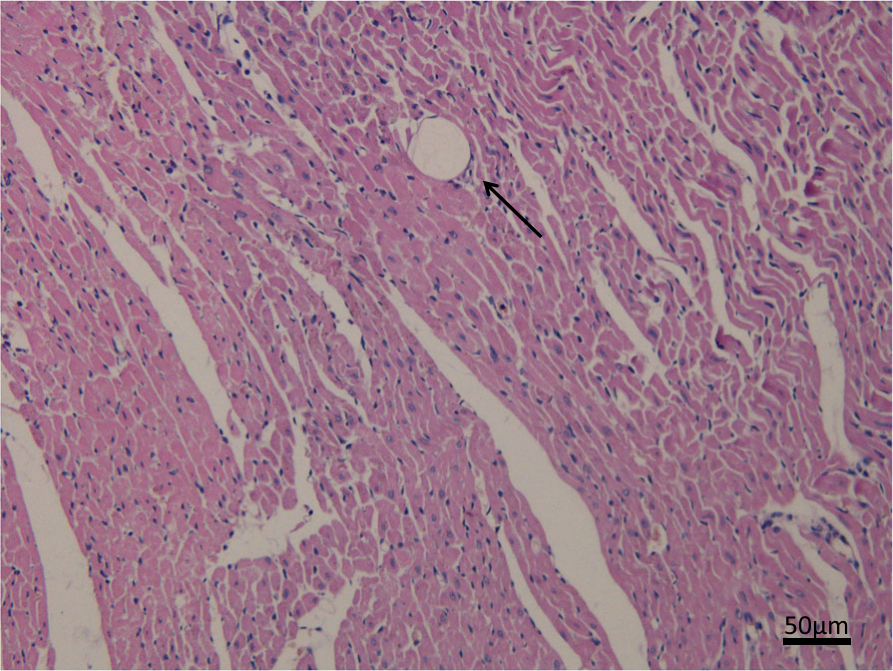
Histopathology of post-CME myocardial microinfarcts (×200), bar = 50 μm. Tissue samples’ HE stain from the CME group. The arrow reveals the exist of a 42-μm microsphere following CME

Six hours after CME modeling, the HBFP staining showed that no obvious myocardial microinfarction in the sham group. Meanwhile, it was remarkably distinct in the CME group and AAV-control (CME) group (*P* < 0.05). As contrasted with CME group, AAV-Nrf2 pretreatment remarkably reduced the micro-infarction area (*P* < 0.05). The proportions of micro-infraction area in the sham, CME, AAV-Nrf2(CME) as well as AAV-control (CME) were 0.03 ± 0.01, 15.20 ± 2.23, 8.28 ± 3.12, 14.90 ± 3.22 respectively (Fig. 1e). The HE staining showed that microspheres were injected into the myocardium (Fig. 2).

### AAV-Nrf2 pretreatment improved the function of heart following CME

The effects of echocardiography represented that the CME group and the AAV-control (CME) group reduced the function of cardiac systolic comparing with the sham group significantly, as showed by the significantly decreased LVEF, FS, as well as CO (*P* < 0.05), while LEVDd was remarkably ascended (*P* < 0.05) at 6 h after CME modeling. Conversely, LVEF, FS, CO significantly increased while LEVDd was remarkably reduced in the AAV-Nrf2(CME) group (*P* < 0.05) (Table 2).

**Table 2:** Coefficient of the function of heart in rats in every group 6 h following CME modeling: the echocardiologic measurement conclusions

Groups	LVEF (%)	FS (%)	LVEDd (mm)	CO (L/min)
Sham	89.25 ± 2.52	42.33 ± 2.56	5.67 ± 2.12	0.232 ± 0.018
CME	52.63 ± 1.78^a^	36.25 ± 3.22^a^	6.51 ± 3.33^a^	0.192 ± 0.033^a^
AAV-Nrf2 (CME)	70.44 ± 3.21^ab^	44.74 ± 1.58^ab^	5.79 ± 1.66^ab^	0.255 ± 0.025^ab^
AAV-control (CME)	52.87 ± 2.12^a^	34.55 ± 2.24^a^	6.48 ± 1.85^a^	0.201 ± 0.011^a^

Data is shown as Mean ± SD

*AAV-Nrf2(CME)* rats injected Nrf2 undergone CME operation, *AAV-control (CME)* rats injected AAV-control undergone CME operation, *CME* coronary microembolization, *LVEF* left ventricular ejection fraction, *FS* fractional shortening, *LVEDd* left ventricular end -diastolic diameter, *CO* cardiac output, *Nrf2* nuclear factor erythroid 2-like, *AAV* adeno-associated virus

^a^ *P* < 0.05 contrasted with sham

^b^ *P* < 0.05 contrasted with CME or AAV control (CME)

### AAV-Nrf2 pretreatment attenuated myocardial damage following CME

Six hours following CME modeling, serum levels of cTnI in rats from the sham group (0.32 ± 0.015 μg/L vs 0.05 ± 0.002 μg/L, *P* < 0.05) were lower than the CME group or the AAV-control (CME) group. What’s more, AAV-Nrf2 pretreatment was involved with reducing cTnI levels (0.19 ± 0.014 μg/L) when contrasted with CME group as well (*P* < 0.05).

### AAV-Nrf2 pretreatment decreased myocardial apoptosis following CME

**Fig. 3:**
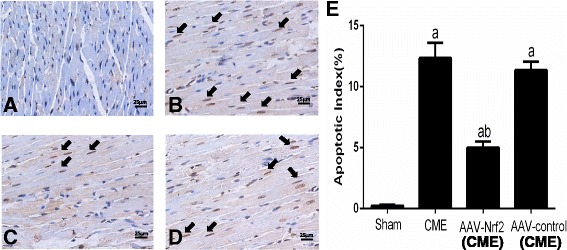
Typical pictures and quantified analysis of myocardial apoptosis in rats from every 6 h after CME modeling: TUNEL staining conclusions. The staining of apoptotic cardiomyocyte nuclei (arrows) are brown (×400), bar = 25 μm. Conclusions of quantified detection revealed that the apoptotic coefficients of myocardium were mainly higher in the CME or the control groups contrasted with those in Nrf2 groups. **a** Sham; **b** CME; **c** AAV- Nrf2(CME); **d** AAV-control (CME); **e** the histogram of the apoptotic index of each group of rats. AAV-Nrf2(CME): rats injected Nrf2 undergone CME operation; AAV-control (CME): rats injected AAV-control undergone CME operation; CME: coronary microembolization; Nrf 2: nuclear factor erythroid 2-like; AAV: adeno-associated virus. Data is presented as Mean ± SD a: *P* < 0.05 contrasted with sham; b: *P* < 0.05 compared with CME or AAV control (CME)

AAV-Nrf2 pretreatment reduced myocardial apoptosis after CME and myocardial apoptosis was measured by TUNEL staining. More TUNEL-positive (yellow-brown) cardiomyocytes can be examined in rats from the CME group when contrasted with the sham group,(*P* < 0.05). In addition, the AAV-Nrf2 remarkably decreased scale of apoptotic cells relatively after CME (*P* < 0.05). The proportions of myocardial apoptotic cells in the sham, CME, AAV-Nrf2(CME) and AAV-control (CME) were 0.23 ± 0.10, 12.34 ± 1.27,5.01 ± 0.50, 11.34 ± 0.72 respectively (Fig. 3e).

### The mRNA levels of Nrf2, HO-1, bax, bcl-2, caspase3 and caspase9

**Fig. 4:**
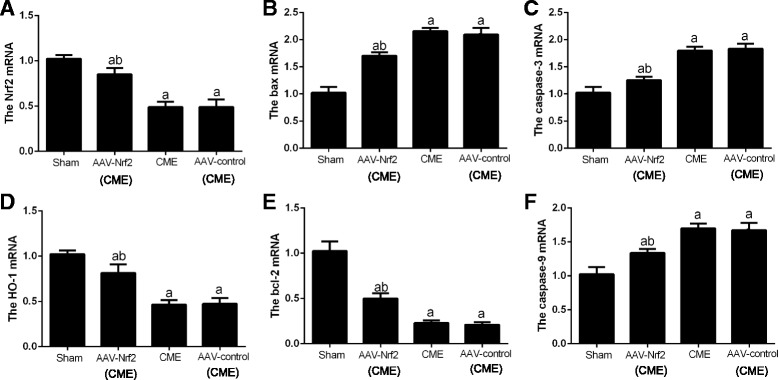
The mRNA expression of Nrf2, HO-1, caspase-3, caspase-9, bcl-2 as well as bax in rats of every group 6 h following CME modeling. AAV-Nrf2(CME): rats injected Nrf2 undergone CME operation; AAV-control (CME): rats injected AAV-control undergone CME operation; **a** Nrf2, **b** bax, **c** caspase-3, **d** HO-1, **e** bcl-2, **f** caspase-9.CME: coronary microembolization; Nrf 2: nuclear factor erythroid 2-like; AAV: adeno-associated virus; Data is shown as Mean ± SD. a: *P* < 0.05 contrasted with sham; b: *P* < 0.05 contrasted with CME or AAV-control (CME)

The mRNA levels of Nrf2 in the sham group and the AAV-Nrf2(CME) group (*P* < 0.05) were remarkably higher than those in the CME group. Moreover, the mRNA Nrf2 (Fig. 4a) level, HO-1 (Fig. 4d) and bcl-2 (Fig. 4e) were significantly lower in the CME and the AAV-control (CME) groups than those in the sham group and AAV-Nrf2(CME) group (*P* < 0.05). In addition, the mRNA levels of bax (Fig. 4b), caspase-3 (Fig. 4c) and caspase-9 (Fig. 4f) were increased significantly in the CME and the AAV-control (CME) groups contrasted with those in the sham group and the AAV-Nrf2(CME) group (*P* < 0.05).

### AAV-Nrf2 pretreatment activated myocardial HO-1 signaling in CME

**Fig. 5:**
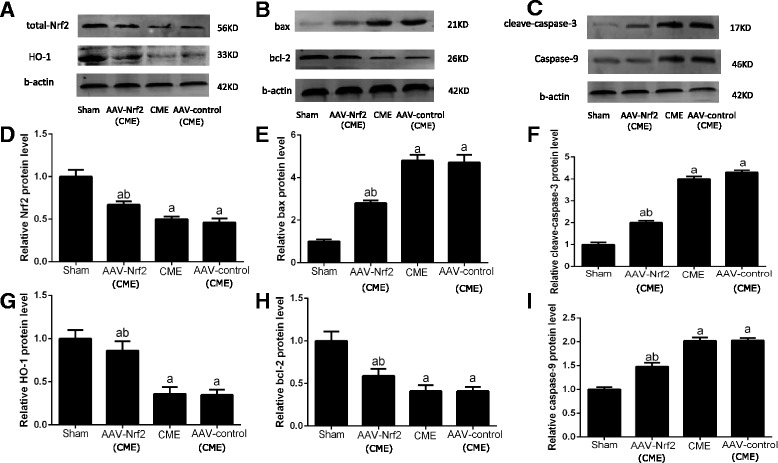
Coronary microembolization reduced cardiomyocyte apoptosis via Nrf2/HO-1 signaling approach. The impacts of CME and AAV-Nrf2 on the protein levels of Nrf2 and HO-1 pathways as well as bax, bcl-2, caspase-3 and caspase-9 in the myocardial tissue in rat 6 h post-CME modeling are as above: the conclusions of the Western blotting detection. AAV-Nrf2(CME): rats injected Nrf2 undergone CME operation; AAV-control (CME): rats injected AAV-control undergone CME operation; **a** the bands of total-Nrf2,HO-1 and b-actin, **b** the bands of bax,bcl-2 and b-actin, **c** the bands of cleave-caspase-3,caspase-9 and b-actin, **d** Nrf2, **e** bax, **f** cleave-caspase-3, **g** HO-1, **h** bcl-2, **i** caspase-9. CME: coronary microembolization; Nrf 2: nuclear factor erythroid 2-like; HO-1: Heme oxygenase-1; AAV: adeno-associated virus. Data is shown as Mean ± SD. a: *P* < 0.05 contrasted with sham; b: *P* < 0 .05 contrasted with CME or AAV-control (CME)

Western blotting exhibited significantly down-adjustment of HO-1 in CME and AAV-control (CME) groups contrasted with the sham group (*P* < 0.05). Nevertheless, AAV-Nrf2 pretreatment enhanced the expression of HO-1 protein (Fig. 5g) and bcl-2 protein (Fig. 5h) contrasted with the CME group or the AAV-control (CME) group (*P* < 0.05), and reduced the expression of cleave-caspase-3 protein (Fig. 5f), caspase-9 protein (Fig. 5i) and bax protein (Fig. 5f) (*P* < 0.05).

## Discussion

In our research, we testified that transfecting Nrf2 attenuated myocardial apoptosis and partially reversed CME, and that Nrf2/HO-1 signaling pathway was concluded in the pathogenesis of CME-induced myocardial apoptosis was saw. The results of this research emphasized the significant part that Nrf2/HO-1 plays in the pathogenesis of CME-connected myocardial dysfunction as well as in the apoptosis. We initially illuminate the part of Nrf2/HO-1 pathway plays in the CME model. These findings could enable us to illustrate the mechanisms by which Nrf2/HO-1 mediates myocardial injury supports our hypothesis that Nrf2 might represent a potential target for therapy.

**Fig. 6:**
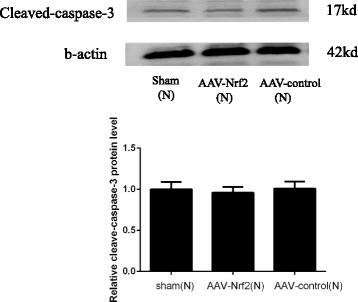
The protein expression of cleave-caspase-3 in the groups of sham (N), AAV-Nrf2(N) and AAV-control (N) of which rats were not undergone operation. Nrf2: nuclear factor erythroid 2-like; AAV: adeno-associated virus. Data is shown as Mean ± SD. #: *P* < 0 .05 contrasted with sham (N); *: *P* < 0.05 contrasted with AAV-control (N)

AAV is a small and nonpathogenic human virus that belongs to the parvovirus family and was originally discovered in the mid-1960s. In the preliminary experiment, we found that there were no statistical difference of cleave-caspase-3 among the groups of rats transfected by AAV-Nrf2 and AAV-control of which rats were not undergone CME and the control rats (Fig. 6).AAV is as a contaminant of cell culture also infected with adenovirus. The high efficiency of in vivo transduction of postmitotic tissues, including heart, brain, and retina, combined with its low immunogenicity, led to the widespread use of AAV as a transfer method for gene therapy in various organs [18–20]. The results and previous studies can be tended to indicate that it has no effect on myocardial apoptosis. Therefore, the rats, which were not undergone CME, were transfected by AAV-Nrf2 and AAV-control groups were not included in further analyses.

**Fig. 7:**
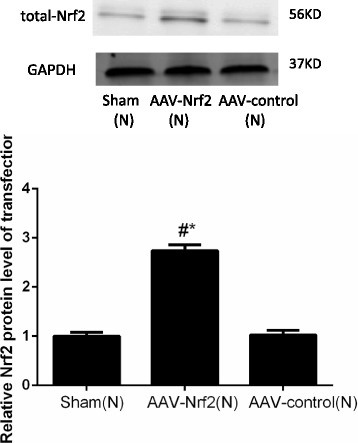
The effects of transfections of AAV-Nrf2 before the operation were checked by Western blotting. The results indicated the AAV-Nrf2 transfected the hearts of rats successfully. Sham (N): rats not undergone operation; AAV-Nrf2(N): rats injected Nrf2 not undergone operation; AAV-control (N): rats injected AAV-control not undergone operation; Nrf2: nuclear factor erythroid 2-like; AAV: adeno-associated virus. Data is shown as Mean ± SD. #: *P* < 0 .05 contrasted with sham (N); *: *P* < 0.05 contrasted with AAV-control (N)

Microcirculation of acute dysfunction and myocardial damage perfusion is a characteristic of CME. Once CME happened, it cannot treat effectively [21]. CME gradually becomes a hot issue. More and more researches are focused on CME. The successful injection of plastic microspheres as the HE staining shown and the micro-infraction of HBFP staining demonstrated the successful establishment of CME model. The CME model was the same as the previously persuasive studies [22, 23]. We upregulated the expression of Nrf2 in rats by transfection of AAV-Nrf2 virus. In the preliminary experiment, the protein expression of Nrf2 in rats that were transfected was higher than the control rats (*P* < 0.05). The result meant the successful transfection of AAV-Nrf2 (Fig. 7).

In the present study, transfection of AAV-Nrf2 can deduce TUNEL-positive cells significantly. TUNEL assay data suggested that the upregulation of Nrf2 could inhibit CME-induced myocardial apoptosis clearly. LVEF, FS and CO of the AAV-Nrf2(CME) group were markedly increased, whereas LVEDd was reduced as contrasted with that of the CME group or AAV-control (CME) group significantly (Table 2). In comparison, the AAV-Nrf2(CME) group had a significant attenuation of levels of cTnI. It was also supported by a significant improvement in cardiac function of AAV-Nrf2(CME) rats.

Apoptosis is a major role in coronary microembolization [24]. The expression Bcl-2 and Bax, which belong to the Bcl-2 family members, is a key part in the cell apoptosis process. It reported that Bcl-2 is an anti-apoptotic protein and then Bax is a pro-apoptotic protein. The proportion of Bcl-2 to Bax is considered as a crucial factor for ensuring the threshold of apoptosis [25]. In addition, it has been found caspase-3 is excited by the apoptotic approach and next processed into excited fragments like cleaved-caspase-3. It’s thought to be an apoptotic coefficient [26]. Caspase-9 has the function to catalyze the activation of caspase-3 [27, 28], whereas caspase-9 is necessary in the cases of apoptotic cell mostly. A large number of studies have suggested that caspase-9 [29] and caspase-3 [30] are involved in the succession of myocardial infarction, also restriction of caspase-9 [31] and caspase-3 [32] can remarkably alleviate ischemia-induced myocardial apoptosis with heart function improvement. In this study, the expression of caspase3, caspase9 and Bax/Bcl-2 significantly increased in CME and AAV-control (CME) groups, and was dramatically reduced in AAV-Nrf2 (CME) group (Figs. 4 and 5). These results indicated that transfection of Nrf2 could decrease the apoptosis of myocardial cells, and improve the cardiac function.

Nrf2 is a gene which has been identified as the especially vital part of Nrf2. The factor of Nrf2 can adjust regular evolving procedures, such as migration, cell growth, apoptosis [33] as well as invasion. Moreover, Nrf2/HO-1 signaling approach is a vital part in the process of apoptosis in the recent studies [34, 35]. Actually, Nrf2 is a crucial modulator of the expression of HO-1 [36].HO is a stress-responsive enzyme which could reduce highly deleterious free heme and produce anti-oxidants, anti-inflammatories, and regulators of apoptosis as well as multiplication, therefore maintaining homeostasis under pathological conditions [37, 38]. There are two distinct mammalian HO isoforms: HO-1 and HO-2. HO-1 is upregulated in the myocardium of rats in response to ischemia-reperfusion injury. HO-1 can also protect cells from apoptosis induced by light [39]. Some researches have clarified that HO-1 expressing is adjusted primarily by Nrf2. Activated Nrf2 has been considered to be a crucial contributor to the expression of HO-1 [40]. Meanwhile, Ning et al. found that down-regulation of miR-142-5p attenuated ischemia/reperfusion injury in hippocampal neurons by promoting Nrf2/HO-1 signaling approach [41]. Liu et al. found that apoptosis was negatively correlated with the Nrf2 and HO-1 expressing, and ultimately affected the expression of cleaved caspase-3 [42]. In our study, mRNA and protein levels (Figs. 4 and 5) of Nrf2 and HO-1 upregulated in AAV-Nrf2(CME) rats and the Nrf2 could reduce the expression of caspase-3 as well as caspase-9 and a ratio of bax/bcl-2 and apoptosis of myocardial cells (Fig. 3), and decrease levels of cTnI, demonstrating activation of Nrf2/HO-1 signaling can improve cardiac function in CME rats. The apoptotic pathways mainly include intrinsic and extrinsic pathways [43]. Some studies have confirmed that Nrf2/HO-1 mainly regulated apoptosis through intrinsic pathway, but relative studies about extrinsic pathway of Nrf2/HO-1 were few [44, 45].In our study, Nrf2/HO-1 could suppress the expression of caspase-9 and caspase-3, and caspase-9 is the key factor of intrinsic pathway and active caspase-3 to induce apoptosis. Nrf2/HO-1 is probably concluded in the process of myocardial apoptosis after CME through the intrinsic pathway. Whether Nrf2/HO-1 can regulate apoptosis through extrinsic pathway, further studies are still needed.

There are likewise some limitations in the current study: Firstly, the conclusions were drawn from the model of rat CME and it was prepared by the injection of the plastic microspheres into the LV. Thus, the present outcome might not be straightforward compared to the results achieved in the patient. Secondly, we only studied the short-term protection of Nrf2. Therefore, how long this protective effect lasts is uncertain. With this in mind, future research needs to be explored in more CME models.

## Conclusions

These findings showed that activating Nrf2/HO-1 signaling can inhibit apoptosis to attenuate coronary microembolization, and also improve the cardiac function. Nrf2/HO-1 signaling pathway is possibly a potential medical goal for treatment associated and it has a chance to be worthy for sick people who are undergone acute coronary syndrome or PCI and improve the long-term prognosis.

## Abbreviations

AAV: Adeno-associated virus
ANOVA: Analysis of variance
CME: Coronary microembolization
HO-1: Heme oxygenase-1
Nrf2: Nuclear factor erythroid 2-like
RT-PCR: Reverse Transcription-Polymerase Chain Reaction
TBST: Tris-buffered saline tween
TUNEL: TdT-mediated dUTP Nick-End labeling
